# Locating suitable habitats for West Nile Virus-infected mosquitoes through association of environmental characteristics with infected mosquito locations: a case study in Shelby County, Tennessee

**DOI:** 10.1186/1476-072X-7-12

**Published:** 2008-03-29

**Authors:** Esra Ozdenerol, Elzbieta Bialkowska-Jelinska, Gregory N Taff

**Affiliations:** 1 Department of Earth Sciences, University of Memphis, Memphis, TN, USA

## Abstract

**Background:**

Since its first detection in 2001, West Nile Virus (WNV) poses a significant health risk for residents of Shelby County in Tennessee. This situation forced public health officials to adopt efficient methods for monitoring disease spread and predicting future outbreaks. Analyses that use environmental variables to find suitable habitats for WNV-infected mosquitoes have the potential to support these efforts. Using the Mahalanobis Distance statistic, we identified areas of Shelby County that are ecologically most suitable for sustaining WNV, based on similarity of environmental characteristics to areas where WNV was found. The environmental characteristics in this study were based on Geographic Information Systems (GIS) data, such as elevation, slope, land use, vegetation density, temperature, and precipitation.

**Results:**

Our analyses produced maps of likely habitats of WNV-infected mosquitoes for each week of August 2004, revealing the areas that are ecologically most suitable for sustaining WNV within the core of the Memphis urban area. By comparing neighbourhood social characteristics to the environmental factors that contribute to WNV infection, potential social drivers of WNV transmission were revealed in Shelby County. Results show that human population characteristics and housing conditions such as a high percentage of black population, low income, high rental occupation, old structures, and vacant housing are associated with the focal area of WNV identified for each week of the study period.

**Conclusion:**

We demonstrated that use of the Mahalanobis Distance statistic as a similarity index to assess environmental characteristics is a potential raster-based approach to identify areas ecologically most suitable for sustaining the virus. This approach was also useful to monitor changes over time for likely locations of infected mosquito habitats. This technique is very helpful for authorities when making decisions related to an integrated mosquito management plan and targeted health education outreach.

## Background

### Previous research

Review of the literature on vector-borne disease modelling led to the conclusion that numerous environmental factors might be critical to WNV dissemination. The modelling approach and variables used in our research are similar to other GIS-based studies that assess environmental risk factors for Lyme disease and Malaria, using information on land use, land cover, forest distribution, temperature, precipitation, soils and elevation [1-8]. For example, a study by Ruiz et al. (2004) in the greater Chicago area evaluated environmental factors such as elevation range, physiographic region, and percentage of vegetation cover to determine human WNV risk during the 2002 outbreak. Cooke et al. (2006) created a landscape-based model and seasonal climatic sub-models to assess risk areas in the state of Mississippi. This model was based on dead bird reports and included several environmental variables such as road density, stream density, slope, soil permeability, vegetation, and climatic factors. They concluded that WNV risk was associated with high road density, low stream density, and gentle slopes. Gibbs et al. (2006) determined that temperature, housing density, urban/suburban land use, and physiographic region are important variables associated with the geographic distributions of WNV in the state of Georgia.

Srivastava et al. (2001) developed a predictive habitat model for forest Malaria vector species using four thematic maps: forest cover, altitude, rainfall, and temperature, and concluded that GIS-based distribution mapping can help pinpoint areas of occurrence at the micro-level, where species-specific, environmentally-friendly control measures can be strengthened. Tachiiri et al. (2006) developed a raster-based model using basic geographic and temperature data to assess WNV risk in British Columbia. Sithiprasasna et al. (2005) classified land use/land cover into five groups: rice paddy, forest, urban, bare land, and water and suggested that classified, remotely sensed data are useful in estimating the distribution of immature and adult mosquito populations in the Republic of Korea. Bian and Li (2006) modelled mosquito larval habitats on the highlands of western Kenya to evaluate if environmental factors such as terrain, surface water, and land use influence the habitats. Using multiple logistic regression, mosquito larval presence was associated with lower elevations, greater wetness, short distances to water, and land use.

Theophilides et al. (2003) used a dynamic monitoring approach using data on human cases and dead bird reports to track the changing spatiality of WNV activity. The Dynamic Continuous-Area Space-Time (DYCAST) system was developed to identify and prospectively monitor high-risk areas for WNV in New York City. DYCAST acts as an 'early warning system' for targeted public health response to WNV.

### WNV in Shelby County, TN

In 2002, the U.S. experienced the largest WNV epidemic ever recorded with 4,156 human cases and 284 deaths [9,10]. Duration and frequency trends of WNV outbreaks observed in Tennessee have corresponded closely to those of the rest of the continental United States. From 2002 to the end of 2006, the state of Tennessee reported 136 human cases, of which 90 cases were in Shelby County [10]. Shelby is the only Tennessee County in which WNV has been present each year since the onset of the epidemic within the state (Table 1); additionally, this county has always reported the highest number of human cases of any Tennessee county.

**Table 1:** Human cases of WNV reported in the United States, Tennessee, and Shelby County. This table is a summary of the WNV infection in humans from the onset of the infection in 1999 until 2006.

Year	National human cases	National human fatalities	Tennessee human cases	Tennessee human fatalities	Shelby County human cases	Shelby County human fatalities
1999	62	7	0	0	0	0
2000	21	2	0	0	0	0
2001	66	9	0	0	0	0
2002	4156	284	56	9	40	6
2003	9862	264	26	1	10	1
2004	2539	100	14	0	12	0
2005	2949	116	17	1	13	1
2006*	4219	161	23	1	15	0

Total	23874	943	136	12	90	8

*cumulative data for 2006 as of 02/06/2007 based on USGS, NWHC [10]

Through discussions with public health personnel in the vector control division of the Memphis and Shelby County Health Department (MSCHD), information was obtained regarding the mosquito fauna of Shelby County and the methods used to prevent the spread of WNV during the years 2001–2004. The mosquito fauna of Shelby County consist of 49 species; twelve of the species are considered common mosquito species (*Aedes albopictus, Ochlerotatus triseriatus, Aedes vexans, Anopheles quadrimaculatus, Culex quinquefasciatus, Culex pipiens, Culex restuans, Culiseta inornata, Uranotaenia sapphirina, Toxorhynchities rutilis septentrionalis, Psorophora howardii, Psorophora ferox*). The principal vectors responsible for transmitting WNV in Shelby County are two mosquito species: *Culex pipiens *and *Culex quinquefaxciatus*.

In 2001, after WNV was first identified in the bird population, the MSCHD began to implement mosquito control operations: the entire city was sprayed every ten days. Spraying the entire city on a ten day schedule during the mosquito season continued in 2002 as well. Avian, as well as mosquito surveillance, started in May 2002. The first positive birds were identified at the end of May 2002, and the first positive mosquitoes were identified in July 2002. The first human case was not reported until late in September 2002.

In 2003, avian and mosquito surveillance started in April, and the first positive mosquitoes were also identified. Larviciding (treating standing water to kill mosquito larvae before they can hatch into adults) was to be carried out throughout the whole county during the summer of 2003, however not all areas were covered due to a personnel shortage. Rather than spraying the entire city on a ten day schedule, mosquito surveillance was used to guide adulticiding (spraying pesticides to kill adult mosquitoes in the air) during the summer of 2003. Locations where trap counts had over 200 total mosquitoes or any positive (WNV-infected) mosquitoes were mapped using Geographic Information Systems (GIS), and adulticiding was carried out in the area of the trap. In addition, adulticiding was carried out in buffer zones of half a mile around human cases.

**Figure 1:**
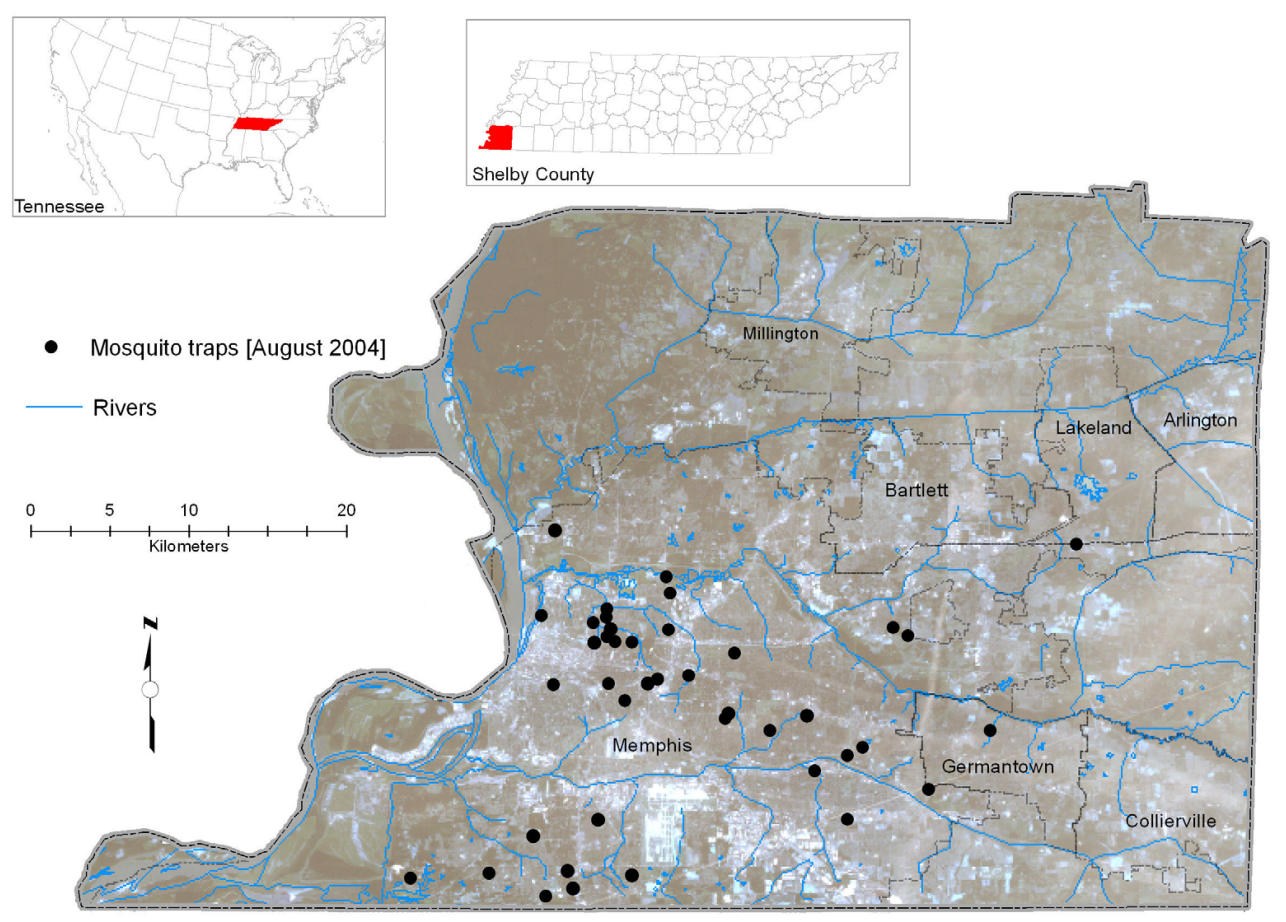
**Study area and distribution of mosquito traps**. This figure shows locations of mosquito traps operated in August 2004 in Shelby County, TN.

**Figure 2:**
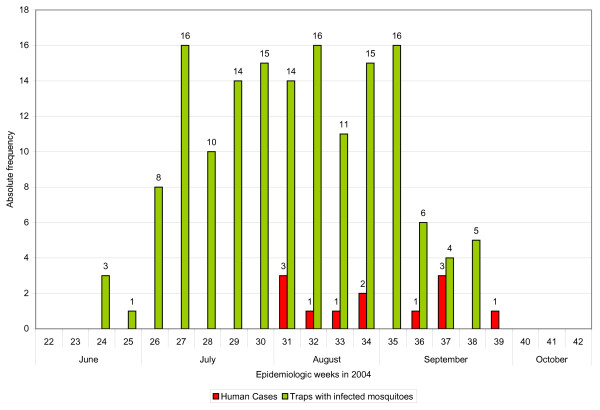
**Human cases of WNV and number of traps with infected mosquitoes by epidemiologic week, Shelby County, TN**. This graph shows the number of WNV-infected humans and the number of traps with infected mosquitoes collected from June through October 2004. Date of onset of illness is used for the reporting of human cases. The epidemiologic week starts on Sunday and ends on Saturday.

In 2004, the first WNV-infected mosquito location was identified in June, and the first human case of WNV was reported in August. All mosquito control activities during the summer of 2004 were the same as in 2003, except that more trap locations were established. Figure 1 shows the locations of the mosquito traps (46) operated in August 2004 in Shelby County. The MSCHD provided us with the human case data with the date of onset of illness. Figure 2 shows the number of reported human cases (onset of illness) and traps with infected mosquitoes by epidemiologic week for the months of June through October, 2004 (note: the epidemiologic week starts on Sunday and ends on Saturday). There is a clear time lag seen in Figure 2 between collection of the first infected mosquitoes of the year and the onset of the first infection in humans. This time lag is expected because once infected mosquitoes are present, it may take time for them to infect humans, and also because of an incubation period in humans. We used mosquito data as an indicator in our research and concentrated on the month of August, since the highest viral activity occurs during this month.

The goal of this project was to analyze environmental data to model habitat suitability for infected mosquitoes. There were three important aspects of the study methodology. First, we mapped the locations of WNV-infected mosquitoes for multiple time points at a countywide scale in a populated urban county. A countywide analysis was best suited for this study since the Memphis and Shelby County Health Department (MSCHD) operates at this geographic level, and it is the responsible organization for mosquito control, allocation of education materials, and other activities which may help in the prevention of WNV and other mosquito-born diseases in Shelby County. Second, we used environmental variables to characterize areas containing the virus. Finally, we developed an innovative GIS-Driven, raster-based technique to construct probability risk maps for areas ecologically most suitable for containing WNV-infected mosquito habitats using Mahalanobis Distance (MD) statistics [11].

## Methods

### Study area and data collection

The study area comprised all of Shelby County, Tennessee. Three types of data were collected: (1) the geographic coordinates of WNV-infected mosquito pools provided by the MSCHD for the year of 2004, (2) environmental data, and (3) socioeconomic data. Data were processed using Erdas IMAGINE [12], Arc View 3.3 [13], and Arc GIS 9.1 [14] software packages.

The selection of environmental variables used in our study was based on evaluation of specific Shelby County mosquito habitat conditions and countywide raster data availability. The environmental variables used were slope, elevation, land use, vegetation density, temperature, and precipitation. We classified these environmental variables as static and dynamic. The static variables included slope, elevation, land use, and vegetation density. The dynamic variables were temperature and precipitation. The slope percent layer was generated from a countywide 30-m Digital Elevation Model (DEM). A 30-m (pixel size of 30 m by 30 m) resolution land use map created by the Tennessee Wildlife Resources Agency through supervised classification was downloaded from the Tennessee Spatial Data Server [15] and cropped to the Shelby county boundary. The land use classes were urban/developed, open water, non-vegetated areas, forested wetland, non-forested wetland, pasture/grassland, row crop, upland deciduous forest, and upland coniferous forest.

To assess vegetation density, we used the Normalized Difference Vegetation Index (NDVI), a commonly used measure of vegetation density, which has previously been shown to be highly predictive of the distribution of disease vectors, including tsetse flies [16] and ticks [17], as well as mosquitoes [18]. The NDVI is a normalized ratio of red and near infrared wavelengths, commonly used to estimate vegetative cover [19]. We derived NDVI from the August 14, 2004, 30-m resolution Landsat TM image using band algebra in Erdas IMAGINE [12].

Climate data of weekly mean temperature and total weekly precipitation at existing weather stations were obtained from the National Climatic Data Center (NCDC) for August, 2004 [20], including each weather station within Shelby County and stations from neighbouring counties in Arkansas, Mississippi, and Tennessee. Data from neighbouring counties were included for the climate interpolation to reduce the influence of edge effects as locations near the edge of a distribution will often have less accurate predictions because there are fewer data points surrounding it [21]. This 'edge effect' can significantly distort the predicted surface at the edge of a study site. August 2004 weekly mean temperature and total weekly precipitation data were interpolated using an inverse distance weighting (IDW) interpolation in Arc View. An evaluation of the residuals determined that IDW [22] performed better than kriging [22,23] for the purpose of this study. The interpolated surface extended beyond the borders of Shelby County, but then was clipped to the desired extent (i.e., the boundary of Shelby County). The interpolated data resulted in two continuous field layers of temperature and precipitation that matched the 30-m resolution grid of other raster layers.

The variables that we used for the socioeconomic analysis were chosen from a review of the literature, in addition to discussions with public health personnel in the vector control division of the MSCHD [24,25,1]. For example, Brownstein et al. (2004) distinguished human case clusters of WNV in New York City based on satellite imagery and census tract data with the use of Kulldorf's (1995) SaTScan spatial statistics software. They found a presence of human cases in areas with an abundance of vegetation, which corresponded to census tracts with low population density. Ruiz et al. (2004) included household income, population age, race, age of housing, housing density, and population density in their analysis to build a descriptive model of the areas with WNV. Their results reveal that a tract in the greater Chicago area is more likely to include at least one case when it has lower population density, is relatively close to bird specimens, comprises a higher percentage of older and white residents and has a higher percentage of housing built between 1950 and 1959.

We collected socioeconomic data from U.S. 2000 Census Summary Tape Files, prepared by the Bureau of Census [26], at the census tract level and included race (white or black), median income, and housing occupation (vacant, owner, or renter). The Shelby County Assessor's Parcel map, subset by census tract boundaries, was used to derive average age of housing for each census tract.

### Methodology

Because of the inherently geographic nature of WNV containment and eradication efforts, some spatial statistical research has been done to understand the aetiology of the disease related to environmental and social factors [8,27-32]. We used an index based on MD statistic to distinguish the favourable habitats for WNV-infected mosquitoes in Shelby County [32], but recognize other mathematical models also could be used, to derive similarity measures based on a set of habitat variables for subsequent mapping by a GIS [33,34].

Mahalanobis distances provide a powerful method of measuring how similar a predetermined set of conditions is to an ideal set of conditions, and can be very useful for identifying which regions in a landscape are most similar to an "ideal" landscape. In the field of vector borne diseases, we might define an "ideal" landscape as that which best fits the niche of some vectors. For example, we may find that mosquitoes typically favour a particular elevation range, slopes of a particular steepness, and perhaps a certain vegetation density. Using Mahalanobis distances, we quantitatively describe the entire Memphis/Shelby County landscape in terms of how similar it is to the "ideal" elevation, slope, land use, temperature, precipitation, and vegetation density for infected mosquitoes. "Ideal" is defined here by the environmental conditions in areas where WNV-infected mosquitoes were actually found in a particular week.

We assume that WNV-infected mosquitoes allocate themselves throughout a landscape, in areas with suitable environmental conditions. We define a multivariate mean and variance of environmental variables for areas where WNV-infected mosquitoes are found for each week of August, 2004. Other favourable habitats are then located for each week of August, 2004 by finding habitats whose environmental variables are similar to those where the WNV-infected mosquitoes were found that week. The level of similarity is determined by the MD statistic, which is essentially a squared distance, normalized by the variances and covariances of a set of variables:

MD = (X-m)^T ^C^-1 ^(X-m),

where X is the vector of environmental data for each pixel, m is the vector of mean values of independent variables for the areas found to contain WNV-infected mosquitoes, C^-1 ^is the inverse covariance matrix of independent variables for the areas found to contain WNV-infected mosquitoes, and T indicates a vector should be transposed. The use of MD statistics "assumes that habitat quality exists as a continuum from highly suitable to unsupportive" [32].

Environmental variables were all converted to grid format and subset to the boundary of Shelby County. Our model was based on six grid layers: elevation, slope, land use, NDVI, temperature, and precipitation. All data were transformed to a common map projection and matched to the same 30-m grid cell resolution. For each week, the MD statistic was calculated for each pixel to determine the similarity of environmental characteristics of that pixel to the pixels where WNV-infected mosquitoes were found that week. Through this method, pixels with favourable habitats for infected mosquitoes were located for each week of August 2004.

**Figure 3:**
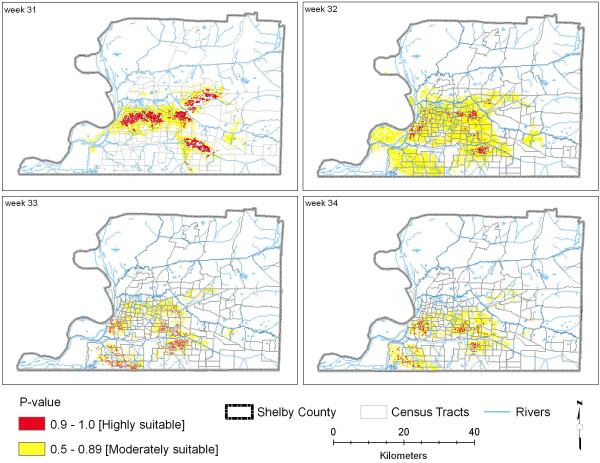
**Habitat suitability based on MD statistic model (Four weeks of August, 2004)**. This map shows moderately and highly suitable habitats based on P-values.

The MD statistic follows an approximate Chi-square distribution with n - 1 degrees of freedom when n explanatory variables are multivariate normally distributed. Following the work of Clark [35] and Farber and Kudmon [36] that characterized suitable species habitats, P-values were determined based on these Chi-square distributions for each pixel, which serves to recode the MD statistic by mapping the Mahalanobis distance values to values between 0 and 1. Maps of the P-values for each pixel in Shelby County were created in the Arc View environment for each week in August. Pixels with highly suitable environments for WNV-infected mosquitoes were identified as pixels whose P-values were greater than 0.9. Pixels whose P-values were between 0.5 and 0.89 were also mapped to show the quantity and spatial distribution of moderately suitable habitats (Figure 3).

**Figure 4:**
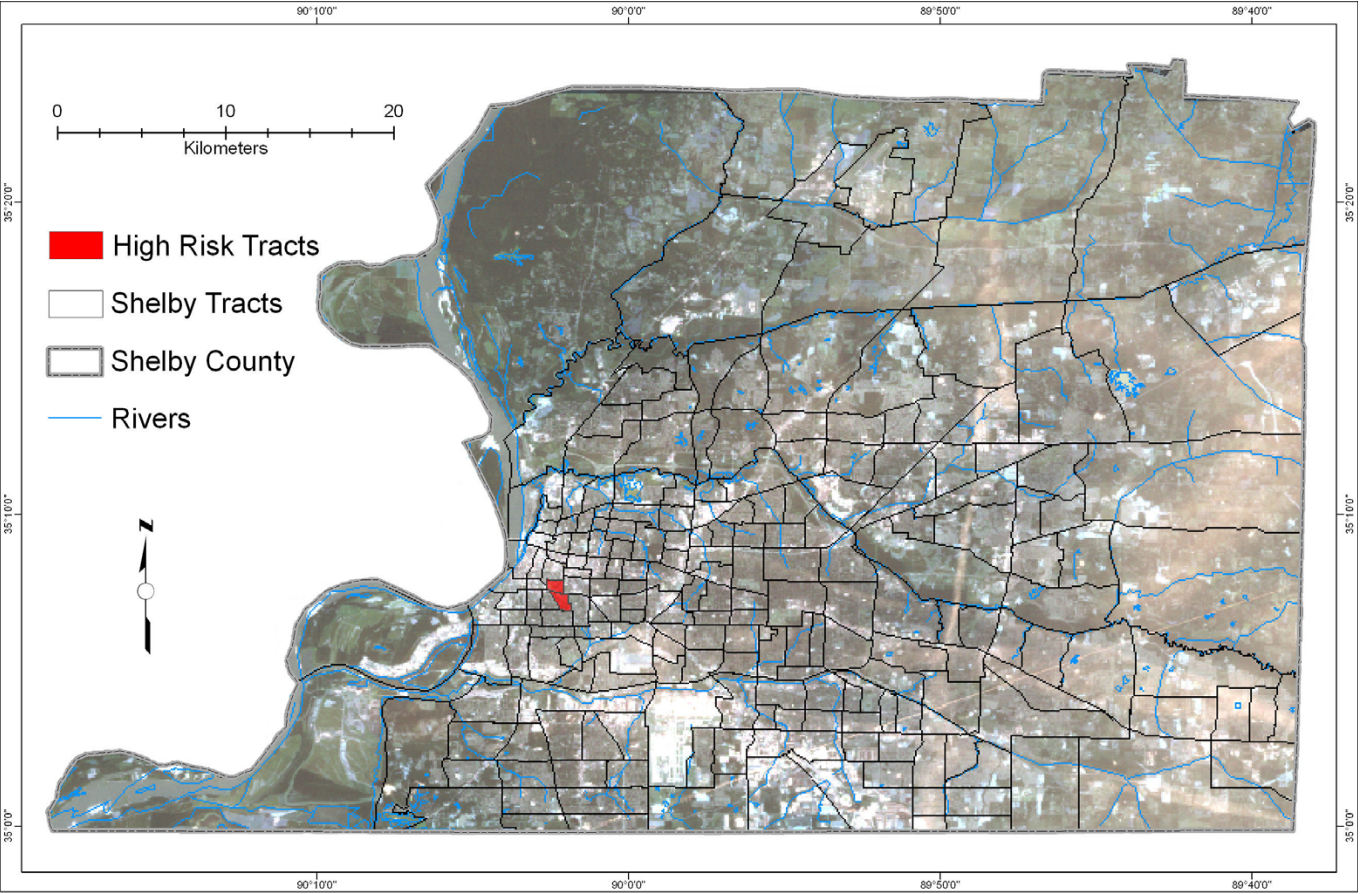
**WNV high risk tracts**. This map shows two tracts in Shelby County, selected based on the intersection of highly suitable habitat areas for WNV-infected mosquitoes for the four weeks of August 2004 based on P-values greater than or equal to 0.9 (P-value ≥ 0.9).

**Figure 5:**
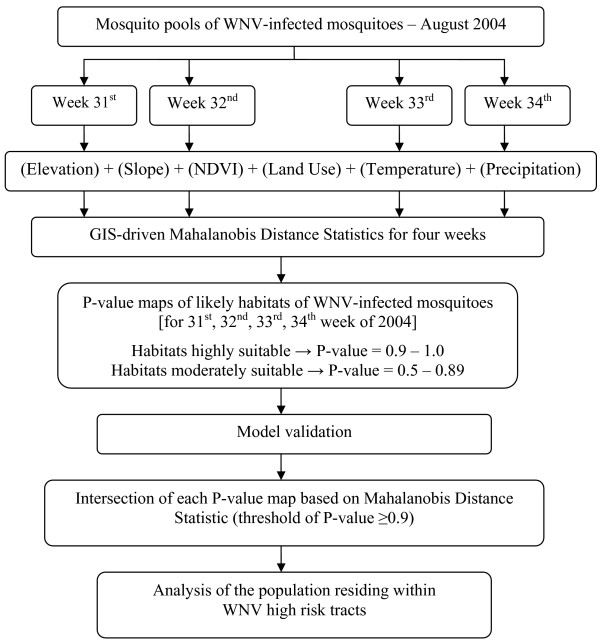
Methodology flowchart.

Additionally, we overlaid the model output of each week and performed a logical intersection of all four weeks depicting areas with high suitability for WNV-infected mosquitoes (P-values ≥ 0.9 for each week). Given its consistent appearance in every week of August, the intersection area is an important area of the predicted potential habitat that merits special attention (Figure 4). We further conducted an analysis of the socioeconomic characteristics of the population residing in this intersection at the census tract level. The methodology development process is detailed in Figure 5.

### Validation Procedure

Since our WNV-positive mosquito sample size for August, 2004 was small (N = 56), we used all data points for creation of the model. The ideal habitats of August 2004 were validated using mosquito data collected in the next month, September (N = 86). The accuracy, sensitivity, and specificity of the model were calculated. Accuracy measures the correct classification rate; sensitivity is defined as the probability of correctly predicting presence, and specificity is the probability of correctly predicting absence [36,37]. The locations were described based on the p-values equal to or higher than 0.5, thus the error matrix used to evaluate the accuracy of the model is presented in Table 2 with frequencies of cases representing the four possible outcomes of a comparison between the predictive map of our model results and the validation dataset. The measures of accuracy, sensitivity and specificity are presented in Table 3. We are more concerned with sensitivity (77%) than specificity (36%) because we want to miss as few potentially positive locations as possible, in order to conduct proper planning for WNV.

**Table 2:** The error matrix to evaluate the predictive accuracy of the model.

		Validation Data Set	
		WNV positive	WNV negative
P-values based on Mahalanobis Distance statistics	More than 0.5	a (24)	b (35)
	Less than 0.5	c (7)	d (20)

Note: The components a, b, c and d, stand for frequencies of cases which were/were not predicted by the model and were present/absent in the validation data set.

**Table 3:** Measures of Model Predictive Accuracy

Measure	Formula
Accuracy (51%)	$\frac{a+d}{n}$
Sensitivity (77%)	$\frac{a}{a+c}$
Specificity (36%)	$\frac{d}{b+d}$

n = a+b+c+d

We used the human cases data set for an additional validation of the model, as humans can be infected with WN virus only via being bitten by an infected mosquito. The appearance of human cases within the ideal habitats of infected mosquitoes could help to validate our model. In the month of August, 2 out of 7 human cases were located in the moderately suitable habitats with P-value 0.5–0.89 and four of the 7 human cases was located in the highly suitable habitats with P-value ≥ 0.9. The one remaining case was also in close vicinity of the moderately suitable habitats (Table 4).

**Table 4:** Validation by human cases. This table shows the number of human cases reported in August and September of 2004 and their locations according to the suitability categories in the MD statistic model

P-value	August	September
0–0.49 (low suitability)	1	1
0.5–0.89 (moderate suitability)	2	2
0.9–1.0 (high suitability)	4	2

Total	7	5

## Results and discussion

We mapped the Mahalanobis Distance as an index of habitat suitability for WNV infected mosquitoes in Shelby County. The Mahalanobis Distance statistics output for each week of August 2004 is presented in Figure 3. We emphasize that the maps represent the similarity of environmental habitat variables at the cell's location relative to the mean vector of the multivariate set of environmental habitat characteristics associated with infected mosquito trap locations for that week. Trap locations are determined based on reported dead bird locations, human or equine case locations, mosquito complaints, known or suspected mosquito breeding sites, and locations of known historical activity. There were 46 traps and 64 mosquito trap collections in August 2004. Mosquito trappings were conducted in rural and uninhabited areas as well as in urban developed areas. 11 (17.2%) of the trap collections were in forested wetlands, which are uninhabited preserved lands. 4 (6.3%) collections were in pasture/grass lands, and 1 (1.5%) was in upland deciduous forest, which is preserved land. 48 (75%) of the trap collections were in urban/developed lands. In this study, we demonstrated that Mahalanobis Distance statistics identified areas ecologically most suitable for sustaining the WNV. This method was also used to monitor habitat change over time, in our case, weekly changes in the month of August 2004.

Results show that ideal environmental conditions for WNV suitable areas tend to be at gentle slopes 0 – 6%, at the elevation lower than 85 meters, temperature = 24–26°C, rainfall = 70 – 75.4 mm/week, and NDVI > 0.3. The highly suitable habitats of the first week of August 2004 encompassed a larger area than the rest of the month (Figure 3: 31^st ^week). Overall, the highly and moderately favourable habitat locations were similar in the first two weeks (Figures 3: 31^st ^and 32^nd ^week), then expanded towards the southwest of the county in the third and fourth weeks (Figure 3: 33^rd ^and 34^th ^week), due primarily to changes in climatic variables. Table 5 presents the number of raster cells and acreage of habitats highly and moderately suitable for WNV-infected mosquitoes for each week of August 2004.

**Table 5:** Number of 30 × 30 meter raster cells (and acres) per habitat suitability class. This table presents the number of raster cells of habitats highly and moderately suitable for WNV-infected mosquitoes.

Class	Week 31	Week 32	Week 33	Week 34
Highly suitable	22,013 (4,896 acres)	9,144 (2,034 acres)	15,455 (3,437 acres)	16,783 (3,732 acres)
Moderately suitable	5,649 (1,256 acres)	1,900 (423 acres)	3,293 (732 acres)	1,462 (325 acres)

Total	242,394 (53,907 acres)			

Our MD statistic model output maps for each week also reveal that the overall areas ecologically most suitable for sustaining the WNV make up much of the core of the Memphis urban area (Figure 3). Housing built before 1940 in the Memphis urban area, especially in Midtown of Memphis, suffered from generally poor drainage, and catch basins were built to reduce backyard and basement flooding. This gives support for theories that the *Culex *mosquito vector prefers highly populated urban and developed land with poor drainage and catch basins with organic material as breeding ground. The assessment of WNV risk to humans cannot be made outside of the urban environment context, since all the human cases resided in these areas. Even though we have address-specific human occurrence data, due to patient confidentiality issues [38], these data were not mapped.

Some areas with high similarity to the mean habitat vector, based on the dynamic and static environmental conditions of each week and infected mosquito locations, remained in the top rankings across the four weeks. We found the intersection of the geographic areas with P-values ≥ 0.9 for each of the four weeks' prediction maps (Figure 4). This was done to find the focal area within Shelby County of environmental conditions well suited for WNV transmission that were consistently present each week. The identification of social factors that characterize this focal area provides insight into human risk and helps to target control and prevention strategies.

The socioeconomic analysis of the two census tracts that include the focal area revealed social drivers of transmission in the county (Table 6). In these high risk tracts, the black population is 98 % of the total population. This is substantially higher than the average percentage of black population in all census tracts of Shelby County (52.6 %). The median incomes for those census tracts are $4,824 and $14,808, which are both below poverty level, according to federal government standards.

**Table 6:** Comparison of socioeconomic variables within high risk census tracts to that of Shelby County overall.

	Black Population %	Vacant Occupation %	Renter Occupation %	Owner Occupation %	Median Income $	Median Year Structure Built
Tract 1	98	27.8	63.4	8.8	4,826	1939
Tract 2	98	23.2	47.9	28.9	14,808	1953
County	52.6	6.8	34.4	55.8	44,197	1971

There are many vacant houses: 27.8% and 23.2%, which is also substantially higher than average for all tracts in Shelby County (6.8%). The percentage of renter occupation (63.4% and 47.9%) is higher than average for Shelby County (34.4%). The higher percentage of vacant and renter occupation are reflected by the very low percentage of owner occupation. The average percent of owner occupation for Shelby County census tracts is 55.8%, which is quite high compared with those two tracts, which are 8.8% and 28.9%. This housing occupation variable was entered into the models because it gives a sense of community commitment to the land. That is, if there is a lot of vacant housing, there is no one to take care of much of the land; if there is a lot of renter-occupied housing, those living there are likely to have less commitment to maintaining the land than in areas with high owner-occupied rates [1,2]. Although all census tracts with these socioeconomic characteristics do not have high P-values, both of those with high P-values had these characteristics.

The socioeconomic conditions in areas that show consistent WNV infections could be reflective of high risk land use decisions such as poor maintenance of rental and vacant properties and a lack of landscaping. The observed heightened risk of infected mosquitoes in lower income areas may have several contributing factors, including landscaping, poor storm drainage systems, lack of screens on windows or air conditioning and backyards with accumulated wet organic material, which, in turn, may be due to community factors such as neighbourhood pride, awareness, and neighbourhood politics.

## Conclusion

This research indicated that the assessment of WNV risk on a county level can be effectively performed using widely available environmental data combined with mosquito surveillance information to support disease monitoring and prediction efforts. By applying GIS-driven MD statistics, we not only identified areas ecologically capable of sustaining the virus, but also could monitor habitat change of infected mosquitoes over four weeks in August 2004. Furthermore, our raster-based approach is not limited by administrative unit aggregation issues [39]. This technique is an effective tool for investigating the spread of vector borne diseases in which vectors are identified only as point locations. Currently in Shelby County, the primary information used to target control measures is based on locations of high mosquito densities, high infection rates, and locations of human cases. Our approach offers the potential for optimization in mosquito surveillance in Shelby County. Locations with high P-values could be targeted for planning field inspections and mosquito spraying. The MD statistic model output gave a detailed distribution of areas likely to contain WNV-infected mosquitoes based on a set of environmental variables at every cell location. Such output can be further analyzed within subsets of the study region, such as recreation areas, census tracts or zip codes. In this study, a detailed assessment of the social factors at the census tract level revealed that human population characteristics and housing conditions such as a high percentage of black population, low income, high rental occupation, old structures, and vacant housing are associated with the focal area of WNV infection cases. Characterizing the environmental factors associated with WNV infection led us to understand the social drivers of WNV infection in Shelby County.

Based on these findings, we recommend an integrated mosquito management plan and targeted health education outreach to reduce the risk of WNV to humans in areas that are environmentally predisposed to harbouring the virus in Shelby County, Tennessee, and elsewhere. Specifically, social factors of race and income are notable in our analysis of Shelby County, and warrant further exploration with qualitative analysis and targeted surveys of the focal area.

While social factors are important when developing a detailed assessment in urban areas, access to real time climatic data is the key for developing a real time early warning system for WNV. Further research in collaboration with MSCHD could provide an online real time mapping of WNV infection and prediction [40,41]. During the active season of the virus, this technique could be applied to provide daily and/or weekly assessments (or other temporal intervals) of the situation based on mosquito surveillance, and this could help public health officials be better prepared to ward off infection cases in humans.

One of the limitations of our study is its focus on infected mosquitoes in general rather than habitat suitability requirements for each specific mosquito species. Different species inhabit a variety of environments and can be found in urban as well as rural settings within specific environmental conditions (*e.g*., catch basins). Lack of data on specific mosquito species infection prevented us from determining environmental variable characteristics that could be based on specific mosquito species habitat conditions.

The quality of our MD statistics output is directly related to the selection of the variables used in the analysis. Good data for dynamic climatic variables are required to quantify the temporal aspects of potential risk of WNV in the county. Spatially and temporally continuous datasets such as temperature and precipitation are important elements of mosquito population dynamics and are commonly modelled through interpolation of values in areas with no observations. We used data from weather stations from nearby regions to interpolate climatic variables to avoid problems with data in boundary areas. For more robust results, new regularly updated monitoring stations in the county and the region are needed. We used a Landsat TM image from August 14, 2004 to derive vegetation density. More images would provide a better temporal estimation and dynamic representation of vegetative cover. The usefulness of selected static variables (elevation, slope, vegetation density and land use) to assess habitat suitability requirements for infected mosquitoes was validated in the literature review and successfully demonstrated in this study. Note that even the static variables need to be updated periodically.

In summary, the technique provided in this study can help better define mosquito control strategies and help regulatory agencies to focus their mosquito-born disease prevention efforts. Finally, it is demonstrated that analyses of environmental variables can be used to ensure a better local understanding of the distribution of WNV, leading to actions that can maintain a safer, healthier population.

## Abbreviations

Abbreviations used in the text, tables or references: Mahalanobis Distance (MD), West Nile Virus (WNV), Geographic Information Systems (GIS), United States Geological Survey (USGS), National Wildlife Health Center (NWHC), Memphis Shelby County Health Department (MSCHD), Digital Elevation Model (DEM), Dynamic Continuous-Area Space-Time System (DYCAST), Normalized Difference Vegetation Index (NDVI), Inverse Distance Weighting (IDW), Landsat Thematic Mapper Data (Landsat TM), National Climatic Data Center (NCDC), and Chi Square (X^2^).

## Competing interests

The author(s) declare that they have no competing interests.

## Authors' contributions

EO acquired data and project funding, was responsible for the conceptual design and administration of the project, participated in and supervised data analysis and interpretation and drafted the manuscript. EB conducted the systematic literature review, carried out the analysis, developed final models, interpreted the results, designed figures and helped to draft the manuscript. GNT supervised statistical analysis and helped to draft the manuscript. All authors read and approved the final version of submitted manuscript.
